# Gravity-Matching Algorithm Based on K-Nearest Neighbor

**DOI:** 10.3390/s22124454

**Published:** 2022-06-12

**Authors:** Shuaipeng Gao, Tijing Cai, Ke Fang

**Affiliations:** School of Instrument Science and Engineering, Southeast University, 2 Sipailou, Nanjing 210096, China; gsp0803@seu.edu.cn (S.G.); fangke@seu.edu.cn (K.F.)

**Keywords:** integrated navigation, gravity matching, K-nearest neighbor, distance weight, Eotvos effect, speed constraint

## Abstract

The gravity-aided inertial navigation system is a technique using geophysical information, which has broad application prospects, and the gravity-map-matching algorithm is one of its key technologies. A novel gravity-matching algorithm based on the K-Nearest neighbor is proposed in this paper to enhance the anti-noise capability of the gravity-matching algorithm, improve the accuracy of gravity-aided navigation, and reduce the application threshold of the matching algorithm. This algorithm selects K sample labels by the Euclidean distance between sample datum and measurement, and then creatively determines the weight of each label from its spatial position using the weighted average of labels and the constraint conditions of sailing speed to obtain the continuous navigation results by gravity matching. The simulation experiments of post processing are designed to demonstrate the efficiency. The experimental results show that the algorithm reduces the INS positioning error effectively, and the position error in both longitude and latitude directions is less than 800 m. The computing time can meet the requirements of real-time navigation, and the average running time of the KNN algorithm at each matching point is 5.87s. This algorithm shows better stability and anti-noise capability in the continuously matching process.

## 1. Introduction

With the development of high-performance inertial measurement units, the inertial navigation system (INS) has been widely applied to navigation. The defect of INS is that the position error would accumulate and diverge with time because of integral operation in the solution process. Other auxiliary methods (such as GPS, astronomy, terrain, geomagnetism, gravity fields, etc.) are needed to correct INS errors during long periods of autonomous positioning [1,2]. The earth’s gravity field has good spatial and temporal distribution characteristics, and gravity anomaly measurement does not require receiving external radiation signals. Gravity-aided navigation is extensively used in navigation, remote-sensing mapping, resource exploration, and other fields because of its all-weather, highly autonomous, unobtrusive, anti-jamming features. This technology is suitable for underwater vehicles and has broad application prospects, which has rendered it an important research object [3].

The gravity-aided inertial navigation system is a technique that makes use of geophysical information. It mainly includes INS, gravimeter, gravity map, and gravity-matching algorithm. The gravimeter measures the gravity anomaly on the movement track in real time. Additionally, the INS provides the sailing information for the gravity-matching algorithm to obtain gravity anomaly data in the gravity map, which is stored in advance. Then, the correlation between the measurement and map data is compared to estimate the real position of the carrier. At last, the matching algorithm outputs the estimated position to the navigation system and provides feedback to the INS on correcting position errors [4]. Currently, the commonly used gravity-matching algorithm mainly consists of the single-point iterative matching algorithm and the sequence iterative matching algorithm. The single-point iterative matching algorithm includes the extended Kalman filter (EKF) and the Sandia inertial terrain-aided navigation (SITAN). This type of matching algorithm is a tracking pattern algorithm, which is suitable for small initial position error. The sequence iterative matching algorithm contains the iterated closest contour point (ICCP), terrain contour matching (TERCOM), and maximum correlation matching [5,6,7,8]. This type of matching algorithm is a search pattern algorithm, which is suitable for large initial position error. In this paper, the maximum correlation matching based on mean square deviation (MSD) and the ICCP algorithm are used for comparison [9,10]. The MSD method is simple, with high calculating speed and easy to implement, which can effectively search the actual position of the carrier; even the INS position error is large after some optimization. The ICCP can also reduce the large error of the INS. It performs iterative calculations on the isoline of gravity field and has high matching accuracy in theory. Because the ICCP uses the equivalent matching method, it is very vulnerable to the influence of gravity measurement noise [11].

Many scholars have conducted a lot of research on the gravity-matching algorithm and achieved a huge number of valuable results. To meet the basic requirement of gravity passive navigation, WANG proposed the operating modes of the integrated navigation system with consideration of the Eotvos effect. Simulation results show that the position error of the system is less than 15% of the goal error over long intervals [12]. OUYANG introduced a robust estimation method to overcome the shortcomings in the gross error detection of SITAN. By setting the adjustment factors, the influence of the abnormal point status matrix and gain matrix on the next location point is reduced, which can maintain the navigation position accuracy within 1 n mile and enhance reliability [13]. XIA improved the traditional gravity-matching algorithm based on maximum correlation by using constraint conditions to eliminate large numbers of interferential data in the searching process, and the proposed algorithm can speed up gravity matching for navigation and improve the orientation precision [14]. JIANG presented a new iterative closest contour point algorithm based on the continuous field with the unique analytic expression. After building the model of searching the closest contour point, the BFGS method of quasi-newton was adopted to locate the closest contour point in the confidence area to improve the matching precision [15]. According to a matched path by searching, tracking, and making decision on a priori gravity map, SUN made full use of the characteristics that INS has, such as high precision, during a short period to realize precise navigation and localization with the aid of gravity. The matched errors under operating sea conditions can be limited to less than one gravity map grid and not influenced by the positional error of INS [16]. In addition, some scholars have applied artificial intelligence algorithms, such as neural networks, support vector machines, and bee colony search to gravity matching and proposed a variety of matching algorithms suitable for gravity-aided navigation to improve the positioning accuracy [17,18,19,20]. These research works improved the positioning accuracy of the gravity-matching algorithm to varying degrees. However, it is limited by the characteristics of gravity field, the resolution and precision of the gravity map, the accuracy of the gravity anomaly measuring instruments, and the positioning, and orientation errors of the INS. The application scope of gravity-aided navigation and the positioning accuracy of matching algorithm can still be improved.

In 1967, Cover and Hart proposed the K-nearest neighbor (KNN) algorithm, which is based on a non-parametric classification model [21]. It is an online machine-learning algorithm that is simple, highly accurate, easy to implement, and has high tolerance for outliers and noise. This algorithm has a sample set for training, and each data sample in the sample set has a classification label and a feature vector. When there is a new data need to be classified, each data sample is compared with the new data by the feature vector. Then, the K most similar data sample is selected by a distance function, and the most frequent classification labels in these samples are deemed as the category of the new data [22]. The KNN algorithm does not need any prior knowledge in the modeling process and does not require adding a specific analytical function to the new data. The relationship between input and output could be obtained by a sufficient amount of representative sample data, and the new data could be directly added into the sample set without retraining. The performance of the KNN algorithm is better than the support vector machine method in the multi-classification problem.

At present, the KNN algorithm is the most widely used fingerprint positioning method in the field of indoor positioning based on the wireless network [23]. Many scholars have contributed useful studies to improve the positioning accuracy of the KNN algorithm; they proposed a lot of new methods of weighting K samples to obtain better results [24]. BI proposed a method of enhanced Gaussian-function-weighted KNN localization. This method obtains a weighted positioning result after creating a standardization process for K-nearest Euclidean distances and distributing the corresponding weights by the Gaussian function. Additionally, the experiments demonstrated that this positioning method can obtain higher robustness and accuracy [25]. Through the fuzzy role processing, SUN obtained the matching degree between the node and the reference nodes, which is used to weigh the K nodes. Additionally, he innovatively proposed the position optimization threshold to determine whether to perform the second fuzzy weighting process [26]. In order to avoid the lack of subjective weighting based on expert experience, XIANG proposed a KNN algorithm based on entropy weight, which is used to adjust the weight proportion of each matching sampling point adaptively based on the internal characteristics [27]. BAI analyzed the unreasonable parts of the common KNN weighted regression algorithm and proposed a method to calculate weight value precisely according to each sample’s position in the whole sample space. This method could describe outliers and sample points, which have important local property [28]. In summary, these weighting methods all use the relationship between the feature vectors in the sample space. The intrinsic properties of sample labels could also be used to determine the value of weights.

The indoor fingerprint positioning method uses the known position data and the received signal characteristic value of the reference points to establish a position and fingerprint database. It then uses the signal strength sequence of the target point to match the fingerprint database and finally solves the position of the target point. This method estimates the position of the target by comparing the received signal strength of the measured point with the existing sample points. It is suitable for gravity field positioning because the principle is similar to the gravity-matching method. A gravity-matching algorithm based on KNN was proposed in this paper. A training sample set was first built in the search range. Then, considering the characteristics of gravity matching, a novel method of weight calculation was designed for integrating samples and introduced the speed of the carrier to improve the accuracy of gravity-aided navigation as a constraint condition in the continuous matching.

Contributions of this paper are summarized as follows:

(1) A novel gravity-matching algorithm based on KNN is proposed. The precision of gravity-aided navigation is improved significantly.
(2) In the KNN sample screening and integration stage, a new method of weighted distance is designed to reduce the effects of the outlier for the first time.
(3) Introducing speed as a condition in the continuous gravity matching, as the first attempt, to limit the mismatch result, which efficiently strengthens the stability of the algorithm.

The rest of paper is organized as follows. In Section 2, the specific process of gravity-matching algorithm based on KNN algorithm is described, including the method of establishment and screening of a sample set, the weight calculation method of K-nearest neighbors, and the speed constraint conditions in continuous matching. The time complexity of KNN algorithm is also analyzed in this section. Subsequently, the performance of the proposed method is verified through an actual test in Section 3. The summary of the experimental results and conclusions are presented in Section 4.

## 2. Materials and Methods

From the perspective of machine learning, gravity-aided navigation could be regarded as a regression process of the vector space of gravity measurement to the vector space of the geographic coordinate system. The algorithm of gravity matching based on KNN, which is proposed in this paper, is combined with the application of gravity-aided navigation to build a dataset and take the average of labels. The specific processes are shown as follows.

### 2.1. Building Dataset of Samples

The INS outputs navigation information, and the gravimeter outputs gravity anomaly measurement in real time. To meet the conditions of gravity matching, sailing data, which include time, location, speed, and gravity anomaly, would be recorded at a fixed sailing distance by the algorithm. Within a radius of R from the location at time T, the longitude and latitude of each grid in this area $S_{T}^{R}$ are chosen as classification labels. Then, a set of sequences of longitude and latitude starting from each label in $S_{T}^{R}$ is obtained by the following equation.

(1) $$P_{i}=A_{\Delta \theta }\left(INS_{N}-INS_{i}\right)+P,\;i=1,\;2,\;\ldots ,\;N$$

where N is the sequence length of gravity matching, P is the classification labels, $INS_{i}$ is the location of INS at the sampling time, $A_{\Delta \theta }$ is the rotation matrix, and $P_{i}$ is the longitude and the latitude of each point in the sequence.

Excluding the initial position error, the real motion trajectory of the carrier should be in the fan area near the indicated track of INS. According to the feature of position error accumulation, two rotation matrices are defined to transform the inertial navigation trajectory in the gravity-matching method based on KNN.

(2) $$\{\begin{array}{c} A_{\Delta \theta }=\left(\begin{array}{cc} \cos\Delta \theta & -\sin\Delta \theta \\ \sin\Delta \theta & \cos\Delta \theta \end{array}\right)\\ A_{\Delta \theta_{0}}=\left(\begin{array}{cc} \cos\;i\Delta \theta_{0} & -\sin\;i\Delta \theta_{0}\\ \sin\;i\Delta \theta_{0} & \cos\;i\Delta \theta_{0} \end{array}\right) \end{array},i=1,\;2,\;\ldots ,\;N$$

**Figure 1:**
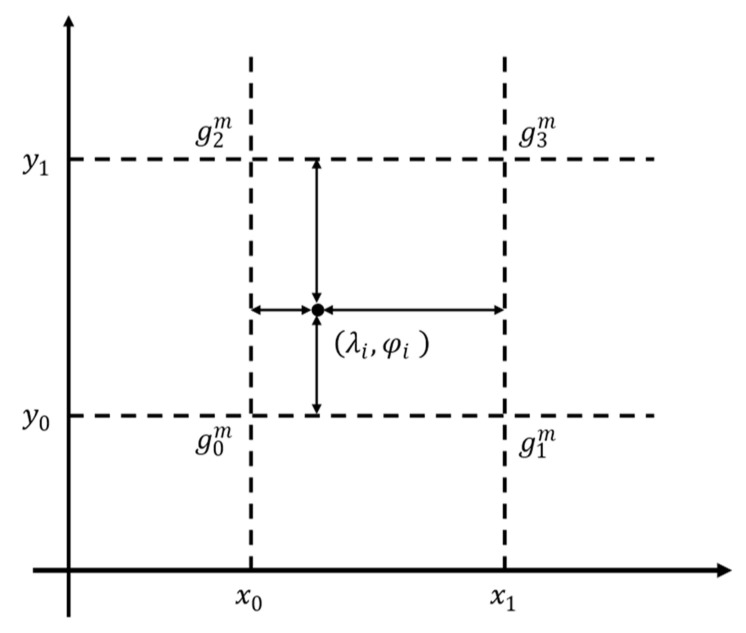
Bilinear interpolation method.

The gravity anomaly of each point in the sequence could be read from the gravity map. Figure 1 shows the principle of using the bilinear interpolation method to obtain gravity anomaly. The formula is defined as below.

(3) $$g_{i}=\frac{\left(y_{1}-\varphi_{i}\right)\left(\left(x_{1}-\lambda_{i}\right)g_{0}^{m}+\left(\lambda_{i}-x_{0}\right)g_{1}^{m}\right)}{\left(y_{1}-y_{0}\right)\left(x_{1}-x_{0}\right)}+\frac{\left(\varphi_{i}-y_{0}\right)\left(\left(x_{1}-\lambda_{i}\right)g_{2}^{m}+\left(\lambda_{i}-x_{0}\right)g_{3}^{m}\right)}{\left(y_{1}-y_{0}\right)\left(x_{1}-x_{0}\right)}$$

where $\left(\lambda_{i},\varphi_{i}\right)$ is the longitude and latitude of the point in the sequence, (x,y) is the longitude and the latitude of the four vertices of the grid, which contains the point, and $g^{m}$ is the gravity anomaly data of these four vertices of the grid.

The gravity anomaly data in the gravity map can be obtained in three ways: gravity inversion from satellite altimeter, airborne gravity measurement, and marine gravity measurement. The satellite measurement can acquire data over a wide range, but the resolution is low. Generally, gravity maps are often subdivided by using the interpolation algorithm [29]. The variation of gravity anomaly is selected as the eigenvector of the sample to decrease the influence of the interpolation error, and the format is shown as below.

(4) $$\Delta g=\left(g_{2}-g_{1},\;g_{3}-g_{1},\;\ldots ,g_{N}-g_{1}\right)$$

**Figure 2:**
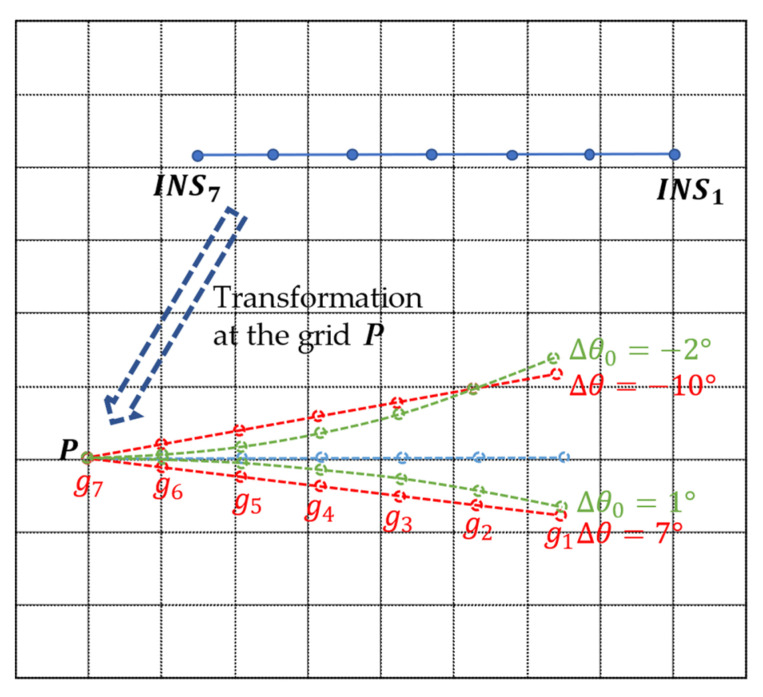
Transform the INS track.

Figure 2 shows the transformation of INS at grid vertices in the search range. With different Δθ and $\Delta \theta_{0}$, a group of track sequences and eigenvectors are generated with the same label. The value of Δθ and $\Delta \theta_{0}$ is determined by the precision of INS, and carrying out the transformation for each label in $S_{T}^{R}$ is helpful for making the sequences as close as possible to the real trajectory of the carrier.

The sample set of $S_{T}^{R}$ is modeled in this step.

### 2.2. Calculating Euclidean Distance

The Eotvos effect will inevitably be produced in the dynamic gravity measurements. While recording the sailing information, the Eotvos effect of the INS trajectory point is calculated in real time by the following formula [30]:

(5) $$E=7.5V\sin\phi \cos\varphi_{INS}+0.004V^{2}$$

where V is the speed of the carrier, and the unit is mile/h in this formula, ϕ is sailing yaw, and $\varphi_{INS}$ is the latitude of the recorded point.

The correction formula for the gravity measurement is

(6) $$G=\tilde{G}-E$$

By transforming the corrected values of gravity anomalies, the test vector with the same dimension as eigenvectors is obtained, and this format also helps reduce the influence of the Eotvos effect.

(7) $$\Delta G=\left(G_{2}-G_{1},\;G_{3}-G_{1},\;\ldots ,G_{N}-G_{1}\right)$$

In the KNN algorithm, the distance between eigenvectors reflects the similarity of labels. The function shown in the following equation defines the distance between two samples.

(8) $$L_{P}\left(x_{1},x_{2}\right)={\left(\overset{n}{\underset{l=1}{\sum }}{\left|x_{1}^{\left(l\right)}-x_{2}^{\left(l\right)}\right|}^{p}\right)}^{\frac{1}{p}}$$

When p equals 1, 2, and infinite, the function above means the Manhattan distance, Euclidean distance, and Chebyshev distance, respectively. The distance between the samples and the test vector is measured by using Euclidean distance in this paper.

### 2.3. Distance-Weighted Method

All samples are sorted in the ascending order of $L_{2}$, and the first K samples are selected. Due to the gravity field varying continuously and randomly, these samples randomly distribute near the real position and may have mismatched points, far from the real position. A weighted average method is used to reduce the influence of outliers in this paper.

Firstly, find out the geometric center of K sample labels.

(9) $$\left(\bar{\lambda },\bar{\varphi }\right)=\frac{1}{K}\overset{K}{\underset{k=1}{\sum }}\left(\lambda_{k},\varphi_{k}\right)$$

Secondly, calculate the reciprocal distance between K sample labels and the geometric center.

(10) $$\eta_{k}=\frac{1}{\sqrt{{\left(\lambda_{k}\cos\bar{\varphi }-\bar{\lambda }\cos\bar{\varphi }\right)}^{2}+{\left(\varphi_{k}-\bar{\varphi }\right)}^{2}}}$$

Next, remove the farthest outlier from the K sample according to the minimal weight data $\eta_{k}$.

Lastly, the weight average result of the rest labels at time T using the following formula.

(11) $$\left(\lambda_{KNN}^{T},\varphi_{KNN}^{T}\right)=\frac{\sum_{k=1}^{K-1}\eta_{k}\left(\lambda_{k},\varphi_{k}\right)}{\sum_{k=1}^{K-1}\eta_{k}}$$

### 2.4. Continuous Gravity Matching

During the voyage, the algorithm records sailing data in real time with a sliding window and obtains the gravity matching result $\left(\lambda_{KNN}^{T+1},\varphi_{KNN}^{T+1}\right)$ at time T+1 by the previous steps. The result of two consecutive moments should meet the speed constraint in spatial location, and the limited condition is described as follows:

(12) $$\{\begin{array}{c} \int_{T}^{T+1}\left(V_{E}-\delta_{V}\right)\leq \lambda_{KNN}^{T+1}-\lambda_{KNN}^{T}\leq \int_{T}^{T+1}\left(V_{E}+\delta_{V}\right)\\ \int_{T}^{T+1}\left(V_{N}-\delta_{V}\right)\leq \varphi_{KNN}^{T+1}-\varphi_{KNN}^{T}\leq \int_{T}^{T+1}\left(V_{N}+\delta_{V}\right) \end{array}$$

where $V_{E}$ is the eastern speed of the carrier, $V_{N}$ is the northern speed, and $\delta_{V}$ is the speed measurement accuracy. Equation (12) indicates that the matching position at time T+1 should be in a rectangular range relative to the matching position at time T. The position and size of the rectangular range depend on the accuracy of the speed measuring instrument. When using the constraint conditions, the unit of parameters should be unanimous. Normally, 1852 m in a straight line is equal to $1^{\prime }$ in longitude and latitude near the equator.

If the result at time T+1 meets the constraint conditions above, the algorithm outputs the matching position as ${\left(\lambda_{kNN}^{T+1},\varphi_{kNN}^{T+1}\right)}^{\prime }$ to the navigation system directly. On the contrary, the matching position at time T will be used to limit the matching result at time T+1 by sailing speed, and the matching position at time T will be output as the following format.

(13) $${\left(\lambda_{kNN}^{T+1},\varphi_{kNN}^{T+1}\right)}^{\prime }=\frac{\left({\left(\lambda_{kNN}^{T},\varphi_{kNN}^{T}\right)}^{\prime }+{\left(\Delta \lambda_{INS},\Delta \varphi_{INS}\right)}_{T}^{T+1}\right)N+\left(\lambda_{kNN}^{T+1},\varphi_{kNN}^{T+1}\right)}{N+1}$$

where ${\left(\Delta \lambda_{INS},\Delta \varphi_{INS}\right)}_{T}^{T+1}$ is the variation of the longitude and latitude of INS from time T to time T+1. Equation (13) shows that there is an iterative process in continuous gravity matching. An estimated position is reckoned according to the last matching result and the position variation in the first. The last result contains earlier results information on gravity matching, so that the estimated position occupies more weight value when averaging it with the matching result at present.

**Figure 3:**
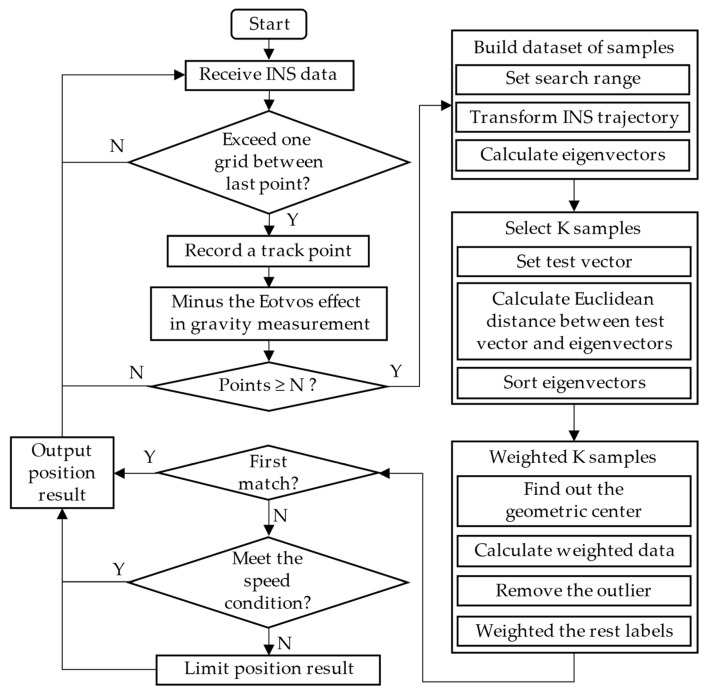
Flowchart of gravity-matching algorithm based on KNN.

The gravity-matching algorithm based on the KNN method proposed in this paper is stated above. It can be seen from the processes of the algorithm that the proposed method is a kind of sequence iterative gravity-matching algorithm. Additionally, Figure 3 shows the flowchart of the algorithm when it is used in the real-time continuous gravity-aided navigation.

The real-time performance of matching algorithms is also a key consideration of gravity-aided navigation. The following is the time complexity analysis result of the proposed algorithm, assuming that there are n grid points in the search radius and transformed INS trajectory m times at each grid point. There are $m{\left(2n+1\right)}^{2}$ sequences created in the search range at the stage of building the sample dataset. The process of calculating the distance between the sample data and the test data is also contained in this stage. Additionally, the program optimizes the sorting and screening of samples, so that the time complexity is $O\left(mn^{2}\right)$ at the stage of building the dataset of samples.

Then, the KNN algorithm applies the method mentioned above to obtain the matching result. The time complexity is $O\left(K^{2}\right)$ while calculating the distance between K samples. Additionally, the time complexity of the other process before output results is O(1). Therefore, in the stage of outputting navigation results, the time complexity is $O\left(K^{2}\right)$.

In general, the total time complexity of the gravity-matching algorithm based on the KNN is $O\left(mn^{2}+K^{2}\right)$, which means the real-time performance is mainly influenced by the search radius and the number of selected samples.

## 3. Experiments and Results

In this paper, INS information and gravity anomaly measurements are selected from an actual ocean experiment. Sailing data are obtained from a strapdown marine gravimeter and a rotation modulation strapdown INS. Through the semi physical simulation platform, the real-time measurement data are simulated to verify the real-time performance and positioning accuracy of the algorithm. The matching algorithm runs on a hardware platform with the main frequency of 2.5 GHz, and the developing flat of the algorithm is VS2010. The gravity anomaly data are downloaded from the global gravity anomaly database in advance. Additionally, the resolution of the database map used in the matching algorithm is $1^{\prime }\times 1^{\prime }$. At the same time, the GPS information received by the carrier is used as the real position to verify the positioning accuracy of the matching algorithm.

### 3.1. Performance Analysis of Application Effect

In order to verify the feasibility of the gravity-matching algorithm based on the KNN method proposed in this paper, the MSD, the ICCP, and the KNN algorithms are applied in the same gravity field, respectively. For the MSD, the method of establishing the sequence to be matched in the KNN algorithm is used to optimize the MSD method. For the ICCP method, the identification range of equivalent points is set by the actual gravity anomaly measurement noise in advance.

**Figure 4:**
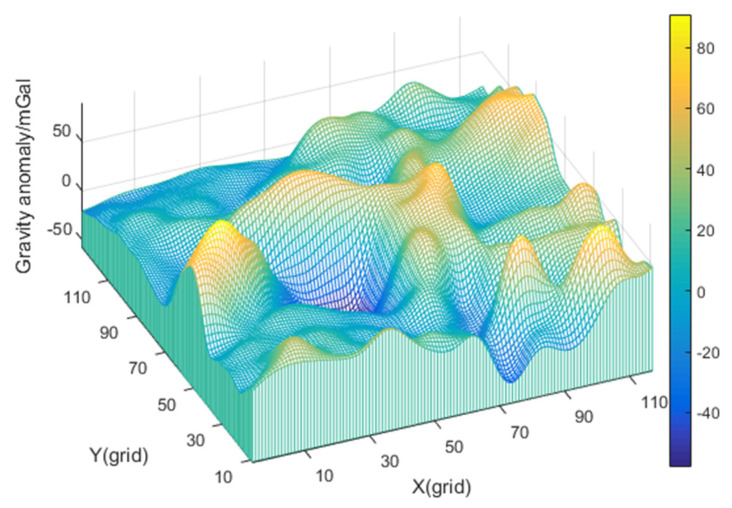
The distribution of gravity field of track 1.

The distribution of the gravity field with obvious features of gravity anomaly is shown in Figure 4. Additionally, the effectiveness of the KNN algorithm will be demonstrated by comparing the matching position errors of the three methods under the same sailing conditions.

When the carrier travels in this area, the gravity-aided navigation system starts to record track points at a particular time and utilizes the gravity-matching algorithm after a while. Table 1 shows the statistics of navigation parameters, gravity anomaly measurement, and features of gravity field in the carrier sailing track.

It can be seen from Table 1 and Figure 4 that the gravity anomaly in this area fluctuates greatly, and the characteristic of the gravity field is obvious. It means this region is suitable for gravity-aided navigation. Based on the actual position of the carrier, the gravity map value at the measurement position is found. Additionally, Table 1 shows that there is a deviation between the gravity measurement value and the gravity map value. This deviation can be regarded as the gravity measurement noise, which is mainly composed of gravity anomaly map error, interpolation error, and gravimeter measurement error. Gravity measurement noise will reduce the positioning accuracy of the matching algorithm.

**Figure 5:**
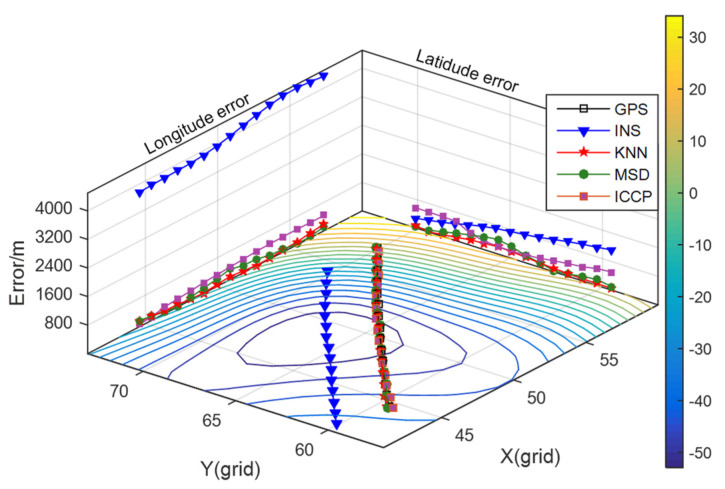
Matching results of track 1.

For these algorithms, the initial parameters are set as follows: the search radius is 0.055°, the step size of interpolation is 0.003°, the initial matching sequence length is 10, the sliding window width is 20 in continuous matching, and the final number of track point is 25. The value of K in KNN is 17. The iteration terminating condition of ICCP is set to a fixed number of 10. The identification range of equivalent points in ICCP is −3~5 mGal. The same speed constraints are introduced into the MSD and ICCP methods in the continuously matching process. The matching results are illustrated in Figure 5, and the statistics of the position errors in INS and matching algorithms are shown in Table 2.

As can be seen from Figure 5, the INS has a large *X*-axis direction error in the beginning. In the subsequent sailing, the *X*-axis direction error gradually decreases, and the *Y*-axis direction error gradually increases. Table 2 shows that these three matching algorithms can effectively find the actual position of the carrier and realize gravity-aided navigation. The positioning accuracy of the KNN algorithm is 32.3% higher than that of the MSD algorithm, and it is 70.1% higher than that of the ICCP method. Under the requirement that the location error of the positioning result is less than half of the grid length of the gravity map, which is equivalent to the straight-line distance of 800 m, the positioning success rate of the three matching algorithms reaches 100% in this area.

It can also be seen from Table 2 that the standard deviation of the positioning error based on the KNN algorithm is less than that of the MSD method and the ICCP method. The KNN method adopts differential method in the sample establishing stage to reduce the influence of the gravity measurement noise. The positioning accuracy and stability of the ICCP is affected by the deviation of the measured value. Due to the randomness and continuity of the gravity field, it may produce outliers in the sample screening stage. The MSD method will be influenced by outliers easily while using the greedy function to screen samples. Additionally, the distance-weighted method proposed in this paper could make use of the spatial location relationship between multiple highly correlated samples. The matching results are not determined by the single sample with the highest correlation, so that it can effectively reduce the impact of the randomness of the gravity field.

In addition, the average running time of the KNN algorithm at each matching point is 5.87 s under the conditions of the hardware platform and current parameter settings of the algorithm. Therefore, the gravity-matching algorithm based on the KNN algorithm proposed in this paper can effectively reduce the error of INS and meet the real-time requirements of gravity-aided navigation.

### 3.2. Performance Analysis of the Anti-Noise Ability

**Figure 6:**
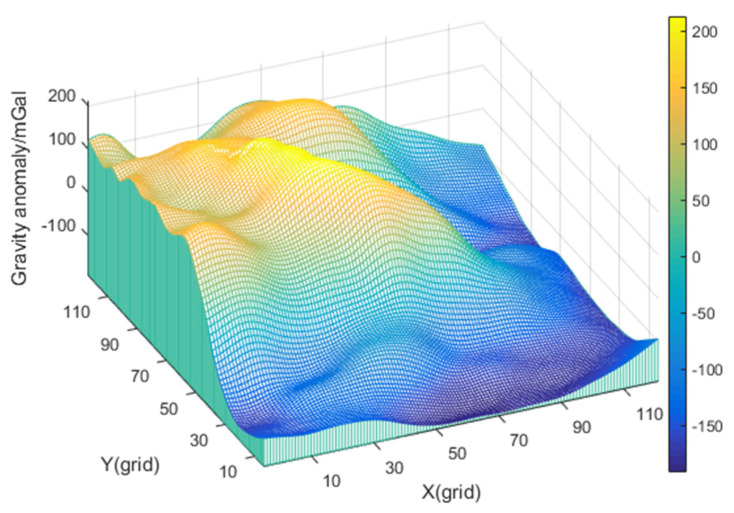
The distribution of gravity field of track 2.

The large deviation between the gravity map and the measurement can easily occur in the area with a large variation of gravity anomaly. Additionally, this deviation is regarded as measurement noise, which will affect the positioning accuracy of gravity-aided navigation. In order to investigate the anti-noise performance of the KNN algorithm, this paper selects a gravity field that has obvious characteristics, and the deviation of gravity anomaly measurement is large. The distribution of the gravity field is shown in Figure 6. The MSD method, the ICCP method, and the proposed algorithm are applied for comparison in this gravity field.

**Figure 7:**
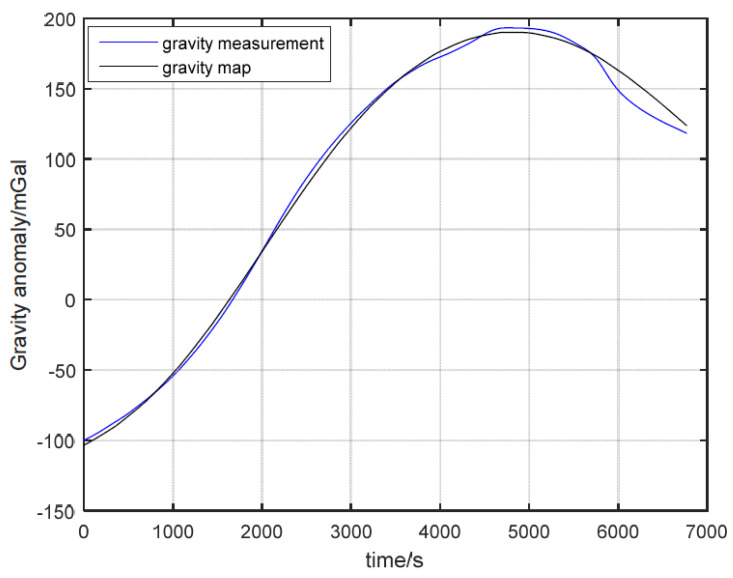
Gravity anomaly measurement and gravity map data.

As can be seen from Figure 6, the variation range of gravity anomaly in this area is large. The gravity-matching algorithm records 25 track points in this area. Figure 7 depicts the variation of gravity anomaly measurement and gravity map value during the voyage. Additionally, Table 3 shows the statistics of navigation parameters, gravity anomaly measurement, and features of gravity field in the carrier sailing track.

Figure 7 reflects that the variation range of gravity anomaly on the trajectory reaches nearly 300 mGal. Theoretically, gravity-aided navigation may achieve a better result in this area. Additionally, the statistical results in Table 3 show that there is a large deviation between the gravity anomaly measurement and the gravity map value in this trajectory, which may lead to false matching results and affect the positioning accuracy.

**Figure 8:**
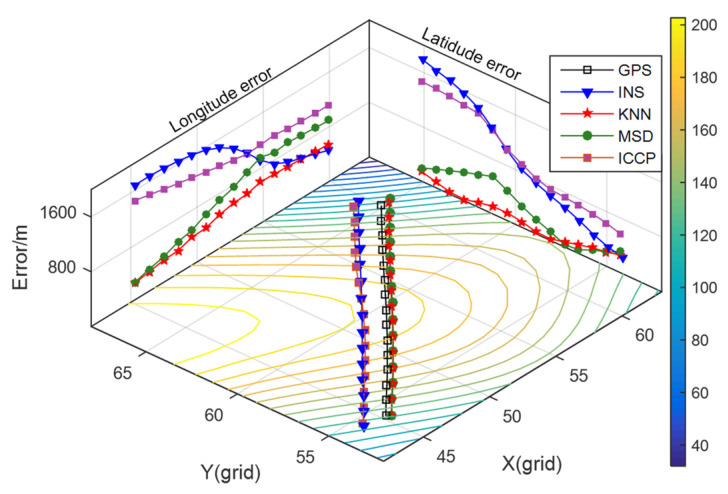
Matching results of track 2.

The MSD algorithm is also used as a comparison with the KNN algorithm in this experiment. Additionally, the parameters of these two algorithms are the same as those set in Section 3.1. The identification range of equivalent points in ICCP is set as −8~6 mGal. The matching results are illustrated in Figure 8, and the statistics of the position errors in INS and matching algorithms are shown in Table 4.

It can be seen from Figure 8 that the positioning errors of INS are less than one grid of gravity map and diverge randomly in the *X*-axis and *Y*-axis direction. According to the criteria of matching failure, the MSD algorithm mismatched in the beginning, while the KNN algorithm maintained a success rate of 100% throughout the whole track. From Table 4, the average positioning error of the KNN algorithm is 34.95% lower than that of the MSD algorithm, but the matching trajectory of the ICCP method is close to the INS trajectory. Because the search range of the equivalent point is wide while gravity measurement noise is large, the grid points near the INS points are regarded as the closest contour points. In the iterative process, the matching track is always near the INS track.

Although the gravity field in this experiment meets the application conditions of gravity-aided navigation, the MSD algorithm is affected when the measurement noise is large, but the KNN algorithm can maintain the matching success rate. The precondition for the ICCP algorithm is that the measurement noise is small, which is difficult to achieve in practical application. This leads to the matching failure of the ICCP algorithm in this experiment.

The gravity fields selected in Section 3.1 and Section 3.2 are all suitable for gravity-aided navigation. With the same experimental equipment and navigation conditions, the main reason for the growth of matching result error is the increase in the deviation between gravity anomaly measurement and gravity map value. This deviation is regarded as random noise of gravity anomaly in the matching algorithm. By the weighted K samples method, the accurate positioning in the case of large random noise is realized, and the anti-noise performance of the gravity-matching algorithm is effectively improved.

### 3.3. Performance Analysis of Adaptation

It can be seen from the experiments in the previous two sections that the gravity-aided navigation is suitable for the gravity field with obvious characteristics. The gravity-aided navigation system selects the adaptation area according to the characteristic value of the gravity field [31]. The positioning accuracy could be effectively improved while using the matching algorithm in gravity adaptive regions. Different gravity-matching algorithms have different adaptabilities to the threshold of the selected adaptation area.

**Figure 9:**
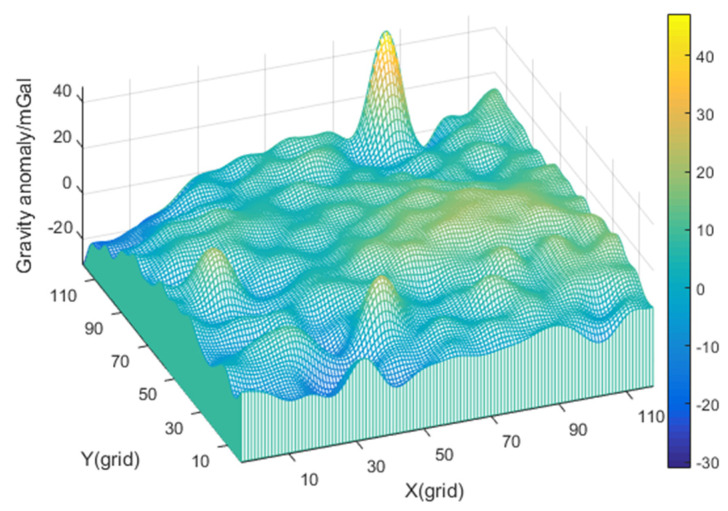
The distribution of gravity field of track 3.

This paper selects a gravity field with weak characteristics, and the distribution is shown in Figure 9. The adaptabilities of the three algorithms to different characteristics are investigated, respectively, by comparing the positioning accuracy.

As can be seen from Figure 9, the gravity anomaly in this area fluctuates gently. The gravity-matching algorithm records 25 track points in this area. Table 5 shows the statistics of navigation parameters, gravity anomaly measurement, and features of gravity field in the carrier sailing track.

**Figure 10:**
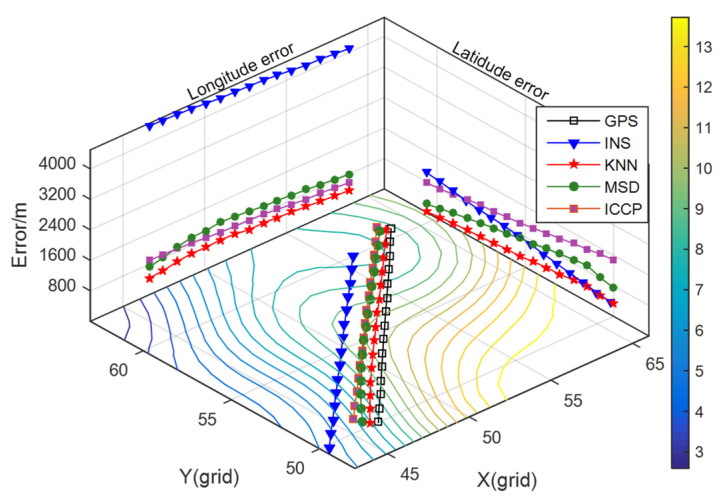
Matching results of track 3.

Table 5 shows that the deviation between the gravity measurement and the gravity map value is small, and the characteristics of the gravity field are relatively weak. The MSD algorithm is compared with KNN algorithm in this experiment as well. The parameters of these two algorithms are the same as those set in Section 3.1. The identification range of equivalent points in ICCP is set as 0~3 mGal. The matching results are illustrated in Figure 10, and the statistics of the position errors in INS and matching algorithms are shown in Table 6.

As can be seen from Figure 10, the positioning error of INS is large. The MSD algorithm and the ICCP method do not successfully match the real position of the carrier. They have the same accuracy level in this area. The matching success rate of the KNN algorithm is maintained at 100%. It means that when using the traditional threshold method to select the matching area, the MSD algorithm and the ICCP algorithm need stricter threshold conditions, while the KNN algorithm is relatively loose. Therefore, the KNN algorithm can adapt to more gravity fields and has wider applicability.

In these three experiments, the positioning errors of the KNN algorithm proposed in this paper are less than 800 m. In the adaptation area, the KNN algorithm can effectively reduce the position error of INS when this error does not exceed the search radius of the matching algorithm. Compared with the MSD algorithm and ICCP algorithm, the KNN algorithm not only has a higher matching success rate but also has a smaller error standard deviation of matching results. It can be applied to more gravity fields and has better stability under long voyage conditions.

### 3.4. Performance Analysis of Speed Constraint

Due to the randomness and continuity of the gravity field, the matching results of each time are discretely distributed near the real position of the carrier. However, the relative position and heading between the adjacent results do not meet the real motion. In this paper, the speed constraint is introduced into the gravity-matching algorithm for correcting the positioning results during continuous matching. The quality of the speed constraint depends on the quality of the velocity measuring instrument.

**Figure 11:**
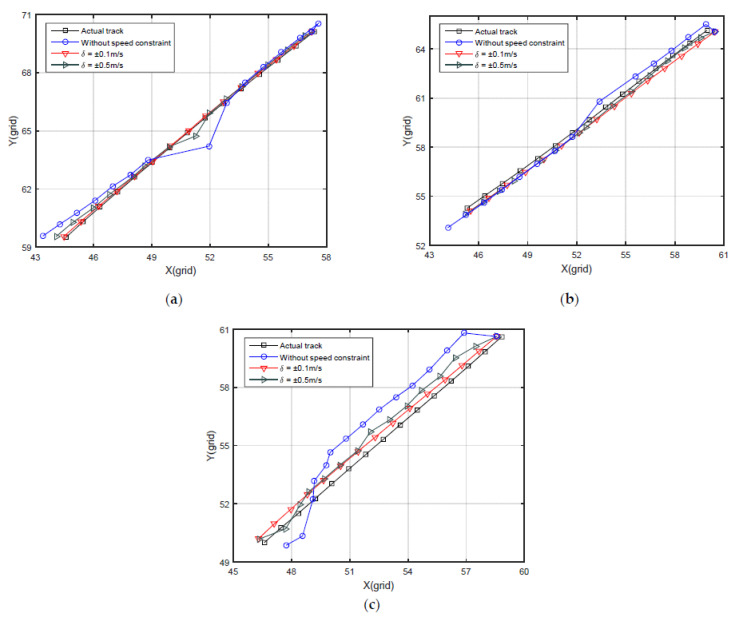
Comparison of speed constraint. (**a**) Matching results of the track in Section 3.1; (**b**) Matching results of the track in Section 3.2; (**c**) Matching results of the track in Section 3.3.

In order to verify the effectiveness of the speed constraint and analyze the influence of instrument accuracy, the KNN algorithm without speed constraint and with different speed measurement accuracy is applied to the first three trajectories in this experiment. The matching results of the three tracks are shown in Figure 11.

As can be seen from Figure 11, the matching trajectories with speed constraint have better convergence and stability in these three results, and their shapes are similar to the actual trajectories of the carrier. After the first matching, the points of matching trajectories without speed constraint are randomly distributed near the real tracks. These points do not correlate with each other and do not conform with the actual sailing orientation and distance. In the case of different speed measurement accuracy, the higher the accuracy of the velocimeter, the stronger the aggregation of the matching results.

By using the speed constraint, the relative position between adjacent matching results meets the actual situation, and the correlation in continuous matching is established. The trajectory formed by the matching points will be stably distributed near the actual position, and the stability of positioning errors is improved. With the speed constraint in continuous matching, the subsequent results can also include the information of the previous matching results, and it also helps reduce the influence of the measurement noise in a single matching process. Therefore, the speed constraint is useful for gravity matching during the long voyage.

## 4. Conclusions

A gravity-matching algorithm based on K-nearest neighbor is proposed and explored in this paper. By establishing a sample dataset in the search range, K-nearest labels were found by Euclidean distance. The matching result is obtained by the weighted average of relative positions between labels, and the speed constraint is introduced into the continuous matching to correct the matching result. The experiment results show that the position error effectively reduces by less than 800 m by the proposed method. The KNN algorithm is more practical than the ICCP algorithm when the measurement noise is large. Compared with the MSD algorithm, the KNN algorithm is not easily affected by outliers when screening samples. Moreover, the algorithm has better stability, anti-noise ability, and wider applicability. Although the computation time in experiments can meet the real-time requirements, the calculation process can still be optimized by the data structure. The factors, which affect matching errors should be discussed in the next step to avoid mismatches in specific areas.

## Figures and Tables

**Table 1:** Information of the sailing track 1.

sailing parameter	total time (s)	8438
	average speed (m/s)	5.19
gravity anomaly measurement deviation (mGal)	mean	1.06
	STD	2.94
characteristic of gravity field (mGal)	max	71.69
	min	−57.49
	longitude roughness (max)	9.91
	latitude roughness (max)	4.93

**Table 2:** Statistics of positioning errors of track 1/m.

	INS		MSD		ICCP		KNN	
	Mean	STD	Mean	STD	Mean	STD	Mean	STD
*X*-axis (m)	4066.6	248.5	121.9	80.3	316.8	123.7	76.7	52
*Y*-axis (m)	720	273.3	175.8	121.9	382.1	140.8	102.3	60.8
Total (m)	4140.7	200.6	221.2	133.5	514.3	125.3	149.6	38.8

**Table 3:** Information of the sailing track 2.

sailing parameter	total time (s)	6768
	average speed (m/s)	6.73
gravity anomaly measurement deviation (mGal)	mean	−1.04
	STD	5.46
characteristic of gravity field (mGal)	max	212.16
	min	−132.42
	longitude roughness (max)	11.97
	latitude roughness (max)	18.34

**Table 4:** Statistics of positioning errors of track 2/m.

	INS		MSD		ICCP		KNN	
	Mean	STD	Mean	STD	Mean	STD	Mean	STD
*X*-axis (m)	1177.4	478.8	694.1	250.7	1223.6	111.4	469.7	124.2
*Y*-axis (m)	978.5	553.2	272.3	153.0	1009.6	341.2	103.2	70.1
Total (m)	1684.4	82.2	759.5	268.7	1616.7	157	494	99.7

**Table 5:** Information of the sailing track 3.

sailing parameter	total time (s)	8456
	average speed (m/s)	5.23
gravity anomaly measurement deviation (mGal)	mean	1.41
	STD	1.46
characteristic of gravity field (mGal)	max	19.26
	min	2.0
	longitude roughness (max)	1.07
	latitude roughness (max)	0.62

**Table 6:** Statistics of positioning errors of track 3/m.

	INS		MSD		ICCP		KNN	
	Mean	STD	Mean	STD	Mean	STD	Mean	STD
*X*-axis (m)	4280	107.9	923.7	100.5	757	104.9	499.5	70.8
*Y*-axis (m)	702	226.9	632.9	228.1	1116.5	226.9	226	118.7
Total (m)	4343.5	70.3	1133.6	170.3	1349.6	146.1	553.9	97.3

## Data Availability

The data presented in this study are available on request from the corresponding author. The data are not publicly available due to privacy reasons.
